# Isolated Psoas Muscle Metastasis From Ampullary Carcinoma: A Report of a Rare Case

**DOI:** 10.7759/cureus.109472

**Published:** 2026-05-23

**Authors:** Fábio Viveiros, Nereida Monteiro, Conceição Monteiro, Cristina Monteiro, Rui Escaleira

**Affiliations:** 1 Department of General Surgery, Local Health Unit of Alto Minho, Viana do Castelo, PRT; 2 Department of Internal Medicine, Local Health Unit of Alto Minho, Viana do Castelo, PRT

**Keywords:** ampulla of vater, ampullary carcinoma, case report, psoas muscle metastasis, skeletal muscle metastasis

## Abstract

Ampullary carcinoma is a rare malignancy arising from the ampulla of Vater. It commonly presents with obstructive jaundice and typically metastasizes to regional lymph nodes, the liver, lungs, and peritoneum. Skeletal muscle metastasis is extremely uncommon, and involvement of the psoas muscle is particularly rare.

We report the case of a 67-year-old man presenting with obstructive jaundice and cholestatic liver function abnormalities. Cross-sectional imaging demonstrated dilation of both the common bile duct and pancreatic duct, consistent with the classic double-duct sign, without a clearly identifiable obstructing lesion. In addition, heterogeneous thickening of the right psoas muscle was observed. Endoscopic retrograde cholangiopancreatography (ERCP) with biopsy confirmed poorly differentiated adenocarcinoma of the ampulla of Vater. Magnetic resonance imaging (MRI) further characterized the psoas lesion, and percutaneous biopsy demonstrated metastatic carcinoma compatible with a biliopancreatic primary tumor.

The patient was diagnosed with metastatic ampullary carcinoma with psoas muscle involvement, and multidisciplinary evaluation also determined the primary tumor to be locally unresectable due to advanced locoregional disease. The patient was therefore referred for palliative systemic chemotherapy.

This case highlights an exceptionally rare metastatic presentation of ampullary carcinoma and emphasizes that atypical muscular lesions in patients with suspected or confirmed malignancy should prompt consideration of metastatic disease and histological confirmation, as accurate diagnosis is essential for appropriate staging and treatment planning.

## Introduction

Ampullary carcinoma is an uncommon epithelial malignancy arising from the ampulla of Vater and accounts for less than 1% of gastrointestinal cancers [1]. Population-based data suggest an incidence of approximately 0.59 per 100,000 persons per year, with a predominance in older adults and in men [2]. Despite its rarity, it remains an important periampullary neoplasm because its anatomic location often leads to earlier symptom onset compared with pancreatic ductal adenocarcinoma [3].

Patients most frequently present with painless jaundice, cholestatic liver test abnormalities, and biliary dilatation on imaging. Other clinical manifestations may include abdominal pain, weight loss, nausea, pancreatitis, or cholangitis [3,4]. In patients with localized disease, pancreaticoduodenectomy remains the standard curative treatment [3]. Histologically, ampullary carcinomas are typically classified into intestinal and pancreatobiliary subtypes, with the pancreatobiliary subtype generally associated with a worse prognosis [4,5].

The most common metastatic sites include the liver, distant lymph nodes, the lungs, and the peritoneum [6]. In contrast, skeletal muscle metastases are rare despite the abundant vascular supply and large mass of skeletal muscle tissue [7-9]. Several hypotheses have been proposed to explain this paradox, including mechanical destruction of tumor emboli by muscle contraction, fluctuations in intramuscular pressure, metabolic factors such as lactic acid accumulation, and local immune surveillance [8,9].

Among skeletal muscles, the psoas is an unusual site of metastasis. Lesions in this location may mimic other conditions such as psoas abscess, hematoma, inflammatory myositis, or primary soft tissue tumors [9,10]. Although unusual metastatic sites from ampullary carcinoma have occasionally been reported, including orbital and bone metastases [11,12], involvement of the psoas muscle appears to be exceptionally rare. In this report, we describe a rare case of ampullary adenocarcinoma with isolated metastasis to the psoas muscle, confirmed by histopathology.

## Case presentation

A 67-year-old man (ECOG 1) presented to the Emergency Department with progressive jaundice (one week) and symptoms consistent with obstructive cholestasis. No weight loss was reported. On physical examination, the patient was markedly icteric. Abdominal examination was unremarkable, without tenderness, palpable masses, guarding, or signs of peritoneal irritation. No palpable gallbladder was identified. The patient denied hip pain, lower back pain, gait disturbance, or limitation of hip flexion related to the psoas lesion. Laboratory evaluation demonstrated hyperbilirubinemia and elevated cholestatic liver enzymes.

Abdominal ultrasound, followed by contrast-enhanced computed tomography (CT), revealed dilatation of both the common bile duct and the main pancreatic duct, producing the classic double-duct sign, without a clearly identifiable obstructing mass. Imaging also revealed heterogeneous thickening of the right psoas muscle (Figure 1).

**Figure 1:**
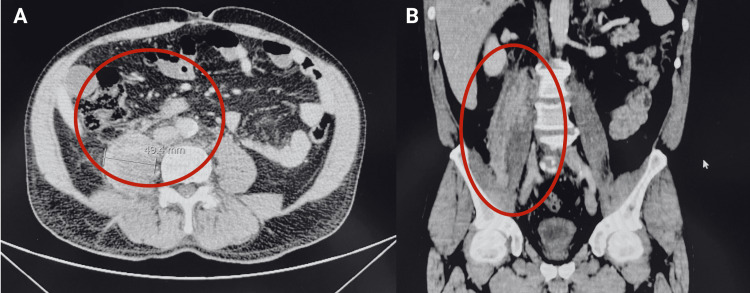
Contrast-enhanced computed tomography (CT) demonstrated biliary and pancreatic duct dilatation and heterogeneous thickening of the right psoas muscle. A) Axial scan; B) Coronal scan.

The patient underwent endoscopic retrograde cholangiopancreatography (ERCP) for biliary decompression. A biliary stent was placed, and biopsies were obtained from the ampullary region. Histopathological examination demonstrated poorly differentiated adenocarcinoma of the ampulla of Vater.

Magnetic resonance imaging (MRI) was subsequently performed for further evaluation of the retroperitoneal abnormality. MRI demonstrated an infiltrative lesion involving the right psoas muscle, raising suspicion for metastatic disease (Figure 2).

**Figure 2:**
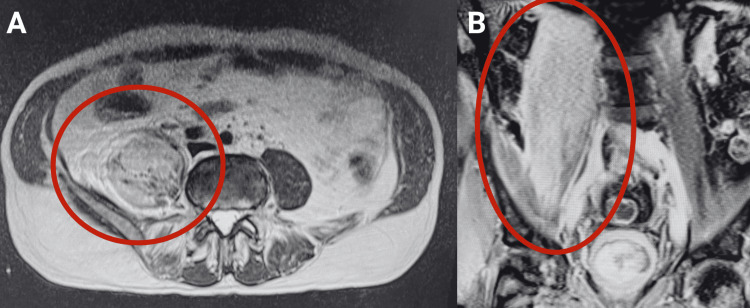
MRI demonstrating infiltration of the right psoas muscle. A) Axial Scan; B) Coronal Scan. MRI: magnetic resonance imaging

Given the nonspecific imaging findings, a percutaneous biopsy of the psoas lesion was performed. Histological analysis confirmed metastatic carcinoma compatible with a biliopancreatic primary tumor.

Based on these findings, the patient was staged as having metastatic ampullary adenocarcinoma with psoas muscle metastasis. In addition, multidisciplinary evaluation determined that the primary tumor was locally unresectable due to advanced locoregional disease (involvement of major arterial structures, including the superior mesenteric artery).

Given the presence of both metastatic disease and a locally unresectable primary tumor, surgical resection was not considered feasible. The patient was therefore referred to medical oncology for palliative systemic chemotherapy.

## Discussion

Ampullary carcinoma is a distinct clinical entity among periampullary malignancies. Compared with pancreatic ductal adenocarcinoma, ampullary tumors often present earlier due to biliary obstruction, which may allow potentially curative surgical treatment in selected patients [3,7]. Prognosis depends on several factors, including tumor stage, nodal involvement, histological subtype, and tumor differentiation [4,5].

The present case illustrates two important aspects of ampullary carcinoma: the possibility of unusual metastatic dissemination and the relevance of accurate resectability assessment.

Resectability of ampullary carcinoma is typically determined using criteria similar to those applied to other periampullary malignancies. Tumors are considered unresectable when there is major arterial involvement, such as encasement of the superior mesenteric artery, celiac axis, or common hepatic artery, or when there is venous invasion of the portal vein or superior mesenteric vein that is not amenable to safe reconstruction [3,7]. Extensive invasion of adjacent retroperitoneal structures may also preclude curative surgery. In the present case, the tumor was considered locally unresectable based on these anatomical criteria.

Skeletal muscle metastases are rare events in oncology, despite the high vascularization of muscle tissue [8]. Several biological mechanisms have been proposed to explain this rarity, including mechanical destruction of tumor cells by muscle contraction, high tissue pressure, metabolic conditions unfavorable to tumor cell growth, and local immune factors [8,9].

When skeletal muscle metastases do occur, they are most commonly associated with lung cancer, colorectal carcinoma, renal cell carcinoma, and lymphoma [9]. The radiologic appearance of muscular metastases is variable and may include focal intramuscular masses, diffuse infiltration, or abscess-like lesions, making diagnosis challenging [8].

Psoas muscle metastases are particularly difficult to diagnose because they may mimic abscesses, hematomas, or inflammatory lesions [10]. In such cases, histological confirmation through biopsy is essential to establish the diagnosis.

Although unusual metastatic sites from ampullary carcinoma have occasionally been described, including orbital or bone metastases [11,12], involvement of skeletal muscle is extremely uncommon. The present case expands the known metastatic spectrum of ampullary carcinoma.

To contextualize this rare presentation, previously reported cases of skeletal or psoas muscle metastases from different primary tumors are summarized in Table 1.

**Table 1:** Selected reports of skeletal or psoas muscle metastases from different tumors.

Study	Primary Tumor	Metastatic Muscle	Diagnostic Method	Key Point
Taira et al. [13]	Renal cell carcinoma	Psoas	Imaging + pathology	Late solitary psoas metastasis reported
Azadeh et al. [14]	Gastroesophageal junction adenocarcinoma	Psoas	Percutaneous biopsy	Rare isolated psoas recurrence
Farraj et al. [15]	Rectosigmoid adenocarcinoma	Iliopsoas	Surgical pathology	Mimicked abscess
Banerjee et al. [16]	Cholangiocarcinoma	Psoas	Imaging + biopsy	Rare biliary metastasis
Bendimya et al. [17]	Colon adenocarcinoma	Psoas	Pathology	Extremely rare muscular spread
Present Case	Ampullary carcinoma	Psoas	Percutaneous biopsy	Very rare metastatic pattern

Recognition of these rare metastatic patterns is important because it influences disease staging and therapeutic decisions [18]. In this patient, the diagnosis of muscular metastasis confirmed advanced disease and supported a non-surgical treatment approach.

Skeletal muscle metastases are generally associated with advanced systemic disease and poor prognosis [8,9]. In ampullary carcinoma, metastatic disease is typically managed with palliative systemic chemotherapy, with survival depending on tumor burden, performance status, and treatment response [3,7]. Although the prognostic implications of isolated psoas muscle metastasis remain unclear due to the rarity of this presentation, its presence likely reflects aggressive tumor biology and advanced disease stage.

## Conclusions

Isolated psoas muscle metastasis from ampullary carcinoma is exceptionally rare. This case highlights the importance of considering metastatic disease when evaluating atypical muscular lesions in patients with suspected or confirmed malignancy.

Histological confirmation remains essential when imaging findings are inconclusive. Early recognition of unusual metastatic patterns may prevent diagnostic delay and ensure appropriate staging and treatment planning.
